# Physiotherapy management of a rare variant of Guillain Barre Syndrome, acute motor and sensory axonal neuropathy (AMSAN) along with COVID-19 in a 35-year-old male -a case report

**DOI:** 10.4314/ahs.v22i3.56

**Published:** 2022-09

**Authors:** Surya Vishnuram, Kumaresan Abathsagayam, Prathap Suganthirababu

**Affiliations:** Saveetha College of Physiotherapy, SIMATS, Chennai, Tamilnadu, India

**Keywords:** Acute motor-sensory axonal neuropathy, Intensive care unit, COVID-19, Gullian Barre syndrome

## Abstract

**Introduction:**

COVID-19 emerged as a novel pandemic with serious illness. Acute motor and sensory axonal neuropathy, a Guillain-Barré syndrome variant also results in ventilator support, and bed-ridden state. Presence of COVID-19 along with GBS will cause serious complications if left untreated.

**Objective:**

To report the effect of physiotherapy in acute motor and sensory axonal neuropathy along with COVID-19 in Intensive care unit.

**Case description:**

A 35-year-old-male with AMSAN, alcoholic hepatitis, and hyponatremia, came with paraparesis, ventilated due to poor oxygen saturation, diagnosed to have COVID-19, reduced muscle power in right wrist extensors, hand grip and diaphragm.

**Method:**

30 minutes physiotherapy session, thrice a day for a period of 4 weeks. The vital signs were taken as a primary outcome measure. Medical Research Council muscle power grading and Hughes functional grading scale were taken as secondary outcomes. All the outcome measures were assessed for 4 weeks.

**Results:**

The 4 weeks of physiotherapy program show significant improvements on health status, muscle power, and functional status of an AMSAN patient with COVID 19.

**Conclusion:**

From the results, it can be concluded that physiotherapy will be beneficial in AMSAN patients with COVID-19 in Intensive care units and further studies have to declare evidence-based practice.

## Introduction

Guillain Barre syndrome (GBS) or Acute Inflammatory Demyelinating Polyneuropathy (AIDP), an autoimmune disorder that involves segmental demyelination and axonal damage following a viral infection; Both antibody and cell-mediated reactions to peripheral nerve myelin were involved and cause nerve cell death, thus regeneration doesn't occur.^1^ About 2 per 100000 population per year experiences GBS, and 20% of them were reported to have variants such as Acute motor axonal neuropathy (AMAN), Acute motor and sensory axonal neuropathy (AMSAN) and Miller-Fisher syndrome.^2^

AMSAN is a rare variant of GBS characterized by an acute muscle weakness, sensory disturbance, pain, diminished tendon reflexes and 20 % of the cases, often occur with cranial nerve involvement and respiratory failure which could end up in tracheostomy, ventilator support, and autonomic involvement such as tachycardia, fluctuating blood pressure, and abnormal respiratory parameters also develops in some cases.^3^

COVID-19 emerged as a serious pandemic in 2019 and numerous people were likely to have been infected.^4^ Combination of GBS along with COVID-19 may result in serious complications if left untreated.^5^ Both Plasmapheresis and Intravenous Immunoglobulin (IVIG) administration are considered to be effective in treating GBS at speedy recovery with ease of administration.^6^, ^7^

Physiotherapy plays a vital role during the course of recovery from COVID-19 by providing respiratory care, muscle strengthening, bed mobility and positioning.^8^ As there was paucity of studies with good level of evidence about role of physiotherapy in GBS patients, there is no structured protocol for physical rehabilitation of AMSAN patient.^9^ There is a need of literature support to rehabilitate AMSAN patients diagnosed with COVID-19. The objective of this study is to report the effect of physiotherapy in a 35 years old male with AMSAN along with COVID-19 and to describe the progression in intensive care unit. Consequently, this study aims to investigate the effects of physiotherapy on vital signs, muscle power of key lower limb muscles, and functional status of an AMSAN patient with COVID-19.

## Case description

A 35-years-old male with complaints of bilateral lower limb weakness, poor oxygen saturation, was bed-ridden with ventilator support. After the clinical examination and investigations, he was diagnosed to have hyponatremia, alcoholic hepatitis, and AMSAN variant of GBS along with features of paraparesis, reduced muscle power in right wrist extensors, hand grip, diaphragm weakness, and poor oxygen saturation. As a routine check-up during the pandemic, Asymptomatic COVID-19 positive report was documented at 2^nd^ day of ICU stay and essential measures were taken. It is noted that 2 other patients in the same ward were also reported to be positive within last week, thus it is unclear that the source of transmission is external or internal. As per the treatment plan, he was isolated to COVID ICU, underwent IVIG in a dose of 2g/ kg of body weight during the initial 5 days of ICU stay and then, referred to the physiotherapy department for further management.

## Subjective Assessment

The subjective assessment from the patient revealed that he was a chronic alcoholic came to the Emergency Department, SMCH, Chennai, India on November, 2020 with complaints of nausea, vomiting, abdominal pain, and lower limb weakness for 2 weeks. While, assessing the past medical history, the patient doesn't possess any systemic or metabolic disorders and he was not under any medication. Based on his personal history, he was an alcoholic for past 3 years, non-smoker and he was a non-vegetarian. Travel history collected from the patient revealed a recent visit to a COVID suspected region nearby his town. His family history, socioeconomic history, and occupational history were unremarkable.

## Objective examination

Vital signs were measured on each session before the therapy and after the therapy. The vital signs on the first day of therapy ranged between (Blood Pressure: 115–140/70–90 mmHg, Temperature: 96–98o F, Pulse Rate: 70–82/min, and SpO2: 96–100%, Respiratory Rate: 28–35/min, in pressure control mechanical ventilator with PEEP 6cmH2O, PIP 10cmH2O, FIO2 50%, and Ti 0.5 sec).

## On observation

Mesomorphic, paradoxical pattern of respiration, patient was intubated with pressure control mechanical ventilator, presented with IV line on left metacarpal vein and urinary condom catheter. Both the lower limbs were extended, abducted, and externally rotated on supine lying. No significant edema, muscle wasting, or postural deformity was present.

## Investigations and provisional diagnosis

The electrolyte report of the patient showed severe hyponatremia (122mEq/L). Grade 1 hepatosplenomegaly was diagnosed by USG abdomen/ pelvis. CSF analysis from lumbar puncture showed elevated protein levels and nerve conduction study resulted in reduced nerve conduction, thus the patient is diagnosed to be AMSAN variant of Guillain barre syndrome. Scrub typhus IgM was detected using IgM ELISA test. On testing the reverse transcription polymerase chain reaction (COVID-19 RTPCR) at 2^nd^ day of ICU admission, the result showed positive asymptomatic COVID-19 infection. The patient also underwent further diagnostic tests for differential diagnosis such as anti-HAV IgM/ anti-HEV IgM for hepatitis, CT-brain for higher centers involvement, and ABG analysis to determine acidity/alkalosis.

## Physical Diagnosis

Bilateral lower limb weakness, right wrist extensor and hand grip weakness, hyporeflexia and hypotonia in both lower limbs, sensory disturbances at varied dermatomes, and diaphragm weakness as a result of AMSAN and COVID-19.

The short term goals were fixed to prevent bed rest/ pulmonary complications, improve muscle power in Lower limbs, right wrist extensors and hand grip, improve oxygen saturation, Enhance airway clearance, Facilitate muscle tone and proprioception in both Lower limbs, Improve sitting balance, and long term goal was to train the patient as wheel chair independent.

## Method

### Outcome measures

The vital signs (heart rate, blood pressure, respiratory rate, and oxygen saturation)^10^, ^11^, ^12^ were noted before and after the therapy, each day from baseline to 4 weeks follow-up as a primary outcome measure. (Table 1)

**Table 1:** Vital signs of the AMSAN patient

Vital signs	Initial assessment	1^st^ week	2^nd^ week	3^rd^ week	4^th^ week
**Heart rate**	143/ min	120–130/min	100–110/min	70–100/min	70–90/ min
**O_2_ saturation**	100% on ventilator	95–100% on 6lits of O2	91–95% on 4 lits of O2	86–92% on 2 lits of O2	92–96% on 4 lits of O2
**Systolic** **Blood** **pressure**	134–140	125–135	126–130	113–120	115–130
**Diastolic** **Blood** **pressure**	82–90	74–90	72–84	76–81	72–76
**Respiratory** **rate**	35/ min on ventilator	24–28/ min on 6lits of O2	25–30/ min on 4lits of O2	28–32/ min on 2lits of O2	22–25/min on 4lits of O2

The Muscle power (flexors, extensors, and abductors of both the hips, flexors and extensors of both the knees, dorsi-flexors and plantar-flexors of both the ankles) were measured using Medical Research Council grading and tabulated in table 2. The MRC is a valid, consistent, and reliable tool^13^, ^14^, where the total scores range from 0 to 5 representing minimal performance and maximal performance, respectively.

**Table 2:** Muscle power of the AMSAN patient

Muscle power MRC grades	Initial assessment	1^st^ week	2^nd^ week	3^rd^ week	4^th^ week
**Hip flexors**	2-	2-	2	2	2+
**Hip extensors**	1+	1+	2-	2-	2
**Hip abductors**	2-	2-	2	2	2+
**Knee flexors**	1+	2-	2	2	2
**Knee extensors**	2-	2-	2	2	2+
**Ankle Dorsiflexors**	0	0	0	0	0
**Ankle Plantarflexors**	0	0	0	0	0

The Hughes (GBS) disability score for functional status were measured from baseline to 4 weeks follow-up and tabulated in table 3. The Hughes disability score (GBSDS) is a reliable and valid assessment tool used to quantify the functional status of GBS patients^15^. Both the MRC grading and GBSDS were taken as secondary outcome measures.

**Table 3:** Functional status of the AMSAN patient

Functional status	Initial assessment	1^st^ week	2^nd^ week	3^rd^ week	4^th^ week
**GBS** **Disability** **scale**	5	4	4	4	4

### Interventions

As the patient might experience fatigue, the physiotherapy program was broken down into 3 consecutive sessions with adequate rest intervals. Thus, the patient was treated for 30 minutes session thrice a day for a period of 4 weeks (a total of 90 mis per day and 5 days per week).

#### 1^st^ week

After framing the long-term goals, standard physiotherapy exercises^16^, ^17^ such as Muscle strengthening Exercises to upper limbs through manual resistance, assisted range of Motion Exercises for lower limbs in eliminated gravity position to retrain the muscles, passive range of motion exercises to lower limb joints and Deep vein thrombosis (DVT) prophylaxis to maintain joint range and peripheral circulation, joint compression to facilitate Tone and Proprioception, and positioning the patient to lateral or supine every 2 hours.^18^, ^19^, ^20^ All the exercises were performed with regular rest intervals for the first 4 days of therapy. On the 5^th^ day, as the patient was weaned off from ventilator and connected to 6 liters of oxygen support through oxygen face mask with 98% saturation, the patient was instructed to start incentive spirometry and breathing exercises from the 5th day and continued till the end of the week.

#### 2^nd^ week

As the vitals became stable in long sitting with 6 liters of oxygen support, the supply was reduced to 4 liters from the 2^nd^ week and the patient is made to sit on the edge of bed with 2 person support for 10 to 20 minutes. Following, the improvement in static and dynamic balance in high sitting, after the 10^th^ day, out of bed mobility to chair with 2 persons shift was started and the duration was progressed day by day from 10 minutes to 120 minutes. There was also improvement in right wrist extensor strength (MRC 2 to 3+) and lower limb muscles (MRC 2- to 2+). The upper limb strength training and assisted Range of Motion exercises to lower limbs in eliminated gravity position were continued with proper progressions in repetitions and sets as Endurance training. The remaining exercises were continued and the improvements in outcome measures were also documented.

#### 3^rd^ week

The RT-PCR after 14 days of admission was taken and the COVID-19 was significantly reduced in the patient's sample. Chair sitting duration increased day by day and the patient made to sit 2 hours at the end of second week. Independent bed mobility and reaching activities in sitting was also initiated from the third week. The patient's dynamic sitting balance improved in-between the third week, thus standing with 2 person support was also started while shifting the patient for 2 to 5 minutes. As the vitals became stable with 4 liters of oxygen support, the supply was reduced to 2 liters from the middle of the 3rd week. But the patient was unable to maintain oxygen saturation (<90%) within 2 hours, thus 4 liters supply was continued with 98% saturation.

#### 4^th^ week

Patient's endurance in lower limbs improved and achieved 30 reps in eliminated gravity position by the end of 3rd week and started manual resisted strength training to both lower limbs in eliminated gravity position. There was also improvement in lower limb muscles (MRC 2+ to 3-). As the patient's vital signs were stable, the patient was planned to shift from ICU to In-patient ward with 4 liters of oxygen supply and the physiotherapy exercises were continued focusing on muscle strength, mobility and functional independence.

## Results

The vital signs were taken as the primary outcome measure, which shows gradual reduction to normal range in respiratory rate, blood pressure, heart rate, and requirement of oxygen support from baseline to end of 4 weeks (Table 1).

On comparing the lower limb muscle power (Table 2), it is well shown that muscle power of hip and knee muscles have improved; and reduction in GBS disability scale (Table 3) when compared from baseline to end of 4th week. From the results, it is proved that 4 weeks of physiotherapy program showed significant improvements on health status, muscle power, and functional status of an AMSAN patient with COVID 19.

## Discussion

The present study aimed to report the effect of physiotherapy on vital signs, muscle power, and functional status of an AMSAN patient with COVID 19. Vital signs were used to assess the patient's health status. The MRC grades were used to assess the muscle power, Hughes Disability scale assessed the functional status. Among various conservative managements, physiotherapy plays a major role in maximizing function and minimizing dysfunction in AMSAN patients.^21^, ^22^

The physical therapy program improved lower limb muscle power in this study similar to the previous study by Fisher et al.^23^ Besides, the results showed improved muscle power in lower limbs, improved static and dynamic balance in sitting, and functional status. Resistance training in this study achieved similar results among AMSAN patients, which has been previously reported by Markvardsen et al and Dimitrova et al.^24^, ^25^

However, COVID-19 appears to be uncommon with GBS26, few cases has been reported in previous studies and recommends COVID test for GBS patients.^27^, ^28^ It was reported that COVID-19 will further reduce the functional ability of GBS patients.^29^ Thus, physiotherapy at early stages of COVID-19 management will facilitate recovery30 and it is also noted that the physical exercise along with the pharmacological treatment influenced the Blood pressure levels, Oxygen saturation, Respiratory rate, and pulse rate. Hence the active exercise program resembles aerobic exercise at a low intensity that is appropriate for the patient with AMSAN, unlike high-intensity muscle exercises.

Physiotherapy has a strong influence on respiratory functions, thus it is essential to restore the physical function effectively after AMSAN.^30^, ^31^ Although, physiotherapy has more beneficial effects on physical rehabilitation in AMSAN patients with COVID-19; the evidence of implying physiotherapy as a standard protocol in clinical practice is lacking in research.

Physiotherapy not only improve physical function in the studied AMSAN patient with COVID-19 but also provide airway clearance and maintain vital signs such as blood pressure, oxygen saturation, and respiratory rate as shown within the current study results.^31^ This single case study assessed the reliable and valid outcomes within the intensive care unit, it is also suggested that future follow-up studies in In-patient wards and out-patient clinics with large sample size will be beneficial to support the evidence-based practice.

## Conclusion

Physiotherapy was found to effectively improve the muscle power and functional status in this AMSAN patient with COVID-19 and alcoholic hepatitis. Therefore, it can be concluded that physiotherapy will be beneficial in AMSAN patients with COVID-19 in Intensive care units and further studies have to declare evidence-based practice. However, low intensity relaxed exercises with tailored repetitions according to the patient's physical ability can improve functional status and restore muscle strength for AMSAN and other neurological conditions.
